# The Effect of an Encapsulated Nutrient Mixture on Food Intake and Satiety: A Double-Blind Randomized Cross-Over Proof of Concept Study

**DOI:** 10.3390/nu10111787

**Published:** 2018-11-17

**Authors:** Annick M. E. Alleleyn, Mark van Avesaat, Dina Ripken, Sinéad B. Bleiel, Daniel Keszthelyi, Ellen Wilms, Freddy J. Troost, Henk F. J. Hendriks, Adrian A. M. Masclee

**Affiliations:** 1 Top Institute of Food and Nutrition, P.O. Box 557, 6700 AN Wageningen, The Netherlands; mark.van.avesaat@mumc.nl (M.v.A.); dinaripken@gmail.com (D.R.); f.troost@maastrichtuniversity.nl (F.J.T.); henk.hendriks5@gmail.com (H.F.J.H.); a.masclee@mumc.nl (A.A.M.M.); 2 Division of Gastroenterology-Hepatology, Department of Internal Medicine, School of Nutrition and Translational Research in Metabolism, Maastricht University Medical Center+, P.O. Box 5800, 6202 AZ Maastricht, The Netherlands; daniel.keszthelyi@maastrichtuniversity.nl (D.K.); e.wilms@maastrichtuniversity.nl (E.W.); 3 The Netherlands Organization for Applied Scientific Research, TNO, P.O. Box 360, 3700 AJ Zeist, The Netherlands; 4 Division of Human Nutrition, Wageningen University, P.O. Box 17, 6700 AA Wageningen, The Netherlands; 5 AnaBio Technologies LTD., Innovation Centre, Carrigtwohill, T45 RW24 Cork, Ireland; sinead.bleiel@anabio.ie; 6 Food Innovation and Health, Centre of Healthy Eating and Food Innovation, Maastricht University, 5911 AA Venlo, The Netherlands

**Keywords:** satiety, encapsulated nutrient mixture, distal release, carbohydrate, protein, overweight, weight management

## Abstract

Activation of the intestinal brake by infusing nutrients into the distal small intestine with catheters inhibits food intake and enhances satiety. Encapsulation of macronutrients, which protects against digestion in the proximal gastrointestinal tract, can be a non-invasive alternative to activate this brake. In this study, we investigate the effect of oral ingestion of an encapsulated casein and sucrose mixture (active) targeting the distal small intestine versus a control product designed to be released in the stomach on food intake, satiety, and plasma glucose concentrations. Fifty-nine volunteers received the active and control product on two separate test days. Food intake was determined during an *ad libitum* meal 90 min after ingestion of the test product. Visual analogue scale scores for satiety and blood samples for glucose analysis were collected at regular intervals. Ingestion of the active product decreased food intake compared to the control product (655 kcal compared with 699 kcal, respectively, *p* < 0.05). The area under the curve (AUC) for hunger was decreased (*p* < 0.05) and AUC for satiety was increased (*p* < 0.01) after ingestion of the active product compared to the control product. Ingestion of an encapsulated protein-carbohydrate mixture resulted in inhibition of food intake compared to a non-encapsulated control product.

## 1. Introduction

The intestinal brake consists of a negative feedback mechanism from distal to more proximal parts of the gastrointestinal (GI) tract and is activated by intraluminal macronutrients and their digestive products. This brake controls not only GI motility and secretion but also food intake and feelings of satiety [1,2,3]. Previously, it was shown that all types of macronutrients are able to activate this intestinal brake and thereby influence eating behavior [4]. While all parts of the small intestine (duodenum, jejunum and ileum) are able to activate this ‘intestinal brake’, the ileal brake is considered to induce the most potent feedback signals in the control of food intake and satiety [5,6]. Therefore, ileal brake activation has emerged as a potential target to reduce caloric intake and, in the longer term, for weight management [7].

Up to now, intestinal brake activation in humans has mainly been studied via intestinal intubation, that is by positioning naso-intestinal feeding catheters in order to infuse nutrients directly into the small intestine [4,5]. These studies have provided clear evidence that infusion of small amounts of either fat or protein or carbohydrates into the distal small bowel reduces food intake and increases satiety. For clinical applications, other less-invasive strategies for macronutrient delivery into the small intestine ought to be developed.

Schellekens et al. and Varum et al., both have successfully designed systems for targeted and site-specific drug delivery in the GI tract [8,9]. A comparable approach can be used for targeted nutrient delivery. Encapsulation of nutrients with a food grade coating provides a barrier against digestion in the proximal GI-tract, and is considered to be a non-invasive alternative for targeted nutrient delivery. Previous studies have employed an oil emulsion product (Fabuless^®^, Olibra^®^) aiming at more distal delivery of fat. These products were based on specific physical-chemical properties of an emulsifier [10,11,12]. The proposed mechanism for Fabuless is the activation of the ileal brake; the emulsified oils delay lipolysis and fat absorption from the proximal GI tract and consequently exposes the distal GI tract to a high intraluminal fat content. Fabuless was shown to decrease food intake and increase satiety in studies by Burns et al. [7,8,9], although this was not confirmed by others [13,14,15,16]. It was hypothesized that the processing of food may have diminished the ability of Fabuless to deliver undigested fat to the distal small intestine [16].

This proof of concept study aimed to investigate the acute effect of an orally administered encapsulated nutrient mixture, targeting at more distal intestinal release, on food intake and satiety compared to a control product that disintegrates in the stomach in healthy overweight subjects. We hypothesized that the encapsulated nutrient mixture more potently induces satiety and reduces food intake during an *ad libitum* meal compared to control condition. We administered two macronutrients: protein (casein) and carbohydrate (sucrose). We chose not to administer fat, since administration of significant amounts of fat in the distal ileum may lead to GI discomfort [17,18]. In addition, fat requires a different encapsulation technique compared to carbohydrates and protein [19].

## 2. Materials and Methods

The study was approved by the Medical Ethics Committee of Maastricht University Medical Center+ and was conducted in full accordance with the principles of the Declaration of Helsinki of 1975 as amended in 2013, and with the Dutch Regulations on Medical Research involving Human Subjects (1998). All subjects gave written informed consent before participation. This trial was registered at www.clinicaltrials.gov as NCT02635659.

### 2.1. Subjects

Subjects were recruited between December 2015 and June 2016. Healthy volunteers were eligible to participate if they were aged between 18 and 65 years and had a body mass index (BMI) between 25 and 30 kg/m^2^. Volunteers were recruited by local advertisements. Exclusion criteria were intake of more than 20 alcoholic consumptions per week, specific medication use or medical history of any relevant disorder or surgery possibly interfering with the study outcomes (as assessed by a physician), known GI symptoms and dieting. All subjects reported to be weight stable for at least 6 months before screening and to be unrestrained eaters (assessed by the Dutch eating behavior questionnaire [20]) and reported not be on a diet during study period. After providing initial information verbally or by email, detailed written study information was provided in case subjects were interested. All subjects had to understand the study procedures before the informed consent was signed. Written informed consent was obtained after an interval of at least 7 days.

The power calculation was based on the decrease in food intake of 136 kcal found in ileal carbohydrate infusion study performed by our group [4]. We expected to find a decrease of 50%, corresponding to 68 kcal. With a standard deviation of 175 kcal, a power of 80% and an alpha of 0.05, a total number of 54 subjects was needed. Anticipating a 10–15% dropout, we included 62 healthy subjects.

### 2.2. Study Design

This double-blind, randomized, controlled crossover study compared the effect of an encapsulated nutrient mixture targeting the distal small intestine with that of an identical control product (nutrient mixture with alginate encapsulation which disintegrates in the stomach). The study product was ingested 180 min after a standard breakfast, to allow the stomach to be emptied before ingestion of the test drinks. Test days were scheduled with a wash out period of 1 week in between to avoid possible carry-over effects. Test days were randomly assigned (by using Research Randomizer, www.randomizer.org). Subjects were randomized to treatment in randomized block designs. The primary randomization factors were gender and age.

### 2.3. Study Products

The active product was an encapsulated nutrient mixture containing 16 g of sucrose (4 kcal g^−1^, van Gilse Automatensuiker, Oud Gastel, The Netherlands), 10 g of casein (energy density: 3.45 kcal g^−1^, Dutch Protein Services, Tiel, The Netherlands) and 2 g of whey protein, which was used to fabricate the microencapsulate (AnaBio Technologies, energy density 112 kcal). The control product contained 16 g of sucrose and 10 g of casein (AnaBio Technologies, energy density 107 kcal). The macronutrients in the control product were encapsulated with a sodium alginate encapsulation system characterized by extrusion of the respective micronutrient using 1.8% (*w*/*v*) sodium alginate, with subsequent drying. Samples were dissolved in a total volume of 80 mL of water before consumption. The drinks were prepared by an independent researcher and offered in a black opaque bottle to blind both the investigator and the participant.

### 2.4. Micro-Encapsulation Procedure

The procedure of preparation of the micro-capsules is described in the patent WO 2016/096931 A1 [21]. All materials were produced according to Good Manufacturing Practice (GMP) guidelines. The micro-encapsulation system utilizes clean-label, food-grade sources of sucrose and casein to generate micron-sized capsules for controlled delivery of native casein and sucrose to the distal small intestine for stimulation of the intestinal brake mechanism. Gastro-resistant micro-capsules were produced when the sucrose and casein formulation was extruded through an apparatus comprising of an outer nozzle concentrically arranged around an inner nozzle, and in which a denatured protein solution is extruded through the outer nozzle and the core-forming casein—sucrose solution is extruded through the inner nozzle. Micro-droplets were instantaneously polymerized in an acidic solution and batches were held at room temperature for max. 1 h under aseptic conditions. This process avoids (1) blockage of sucrose—casein solution in the concentric nozzle and (2) flow discrepancies during the encapsulation process, which would affect encapsulation efficiency of the sucrose-casein micro-capsules.

### 2.5. Characterization of Micro-Encapsulates

#### 2.5.1. Size Distribution and Drying Effects

According to light microscopy, micro-beads demonstrated diameters of approximately 200 μm with a narrow range size distribution (±1.2 μm). Laser diffractometry was also incorporated and confirmed a D (ν, 0.9) value for micro-encapsulates, revealing a diameter of 201.7 ± 0.90 μm and 183.42 ± 0.90 μm, pre- and post-drying respectively.

#### 2.5.2. Stomach Incubation and Strength of Micro-Encapsulates

Strength of micro-beads was analyzed as a function of gastric incubation time in vivo (pH 1.2–1.4; 37 °C). No difference in micro-bead strength was reported for stomach incubation and enzyme-activated stomach conditions did not significantly change micro-bead strength. Tensile strength of micro-beads remained unchanged with no reported leakage or loss of encapsulated casein, pea protein or sucrose. After 180 min of gastric incubation, encapsulated casein, pea protein and sucrose microencapsulates maintained a high tensile strength 52.03 ± 1.27 nN, 60.31 ± 0.27 nN and 58.23 ± 0.12 nN, respectively, to support a robust and high-yielding encapsulation systems for macronutrients.

#### 2.5.3. Intestinal Incubation and Degradation

Transit time of microencapsulates was investigated during in vivo trials and they observed that 35 min after oral ingestion there was no degradation of the micro-encapsulates in the duodenum. The maintenance of micro-encapsulate integrity in small intestinal fluids was tested and degradation was not evident. As time progressed, the capsulate membrane gradually degrades to release to mononuclear core material [21].

### 2.6. Protocol

**Figure 1:**
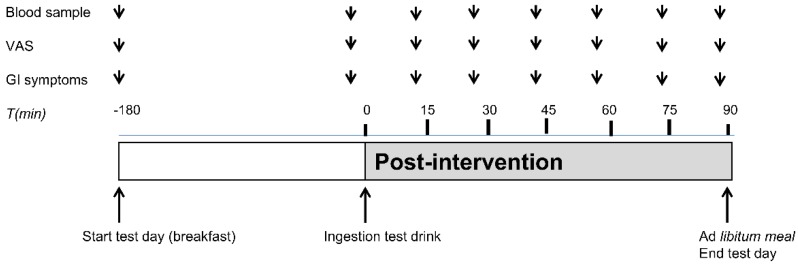
Timeline of the test day. A test drink (encapsulated nutrient mixture or control) was ingested 180 min after finishing the breakfast. Blood samples, VAS scores for satiety and GI symptom scores were collected at regular intervals as indicated.

On each test day, subjects arrived at 745 h, after a 10-h overnight fast, at the Metabolic Research Unit at the Maastricht University Medical Center+. Subjects were instructed to abstain from heavy exercise and consumption of alcoholic beverages the evening before the test day and to consume the same habitual meal on the evening before each test day. On both test days, an intravenous cannula was placed in a forearm vein for collection of blood samples. At 800 h, a fasted blood sample was taken and Visual Analogue Scale (VAS) scores for satiety, hunger, fullness, desire to eat and GI symptoms were obtained. Subsequently, a standardized breakfast meal (identical on both test days), consisting of a whole grain sandwich with cheese (Jumbo Supermarkt; energy 245 kcal per served portion, 13.05 g protein, 5.1 g carbohydrates, 1.75 g lipid) and a 200 mL water was consumed by each participant. Completion of the breakfast meal was considered as t = −180 min. (Figure 1). The subjects were allowed to drink 200 mL water until t = −60 min. 180 min after breakfast intake, subjects ingested the micro-encapsulated nutrient mixture or the control product in randomized order on different test days (t = 0 min). The participant was instructed to ingest the test drink within one min. At 90 min after the intake of the test drink, volunteers received a standardized *ad libitum* lunch meal (Lasagna Meal Jumbo Supermarkt; energy density per 100 g: 144 kcal, 9.0 g protein, 15.2 g carbohydrates, and 4.9 g lipid) (t = 90 min). The *ad libitum* lunch meal was offered in excess (approximately 1 kg of pasta meal) and subjects were instructed to eat until comfortably satiated. *Ad libitum* food intake was assessed by weighing the pasta meal that was not consumed and subtracting that from the weight of the provided meal. After finishing the lunch meal, the test day was finished and subject were allowed to return home.

### 2.7. VAS Scores for Satiety, Fullness, Desire to Eat, Hunger, and Evaluation of GI Symptoms

Satiety, hunger, fullness and desire to eat were measured using VAS scores (0–100 mm) anchored at the low end with the most negative of lowest intensity feelings (extremely unpleasant, not at all) and with the opposing terms at the high end (extremely pleasant, very high, extreme) [22]. GI symptoms were evaluated using a questionnaire which addresses complaints such as nausea, bloating, headache, and other symptoms. Symptoms were scored on a 4-point scale with grade 0 representing ‘not present’ to 3 ‘strongly present’. Subjects were asked to mark the VAS and GI symptom scores before breakfast consumption. This was considered as t = −180 min. Subjects also marked the VAS and GI symptom scores before ingestion of the test drink. After ingestion of the test drink (between t = 0 min and t = 90 min), the VAS and GI symptoms were scored at 15 min intervals until starting the *ad libitum* lunch meal.

### 2.8. Blood Sample Collection

Venous blood samples were drawn before breakfast (fasted) and before ingestion of the test drink (t = 0 min). After ingestion of the test drink, blood samples were obtained every 15 min until the start of the *ad libitum* meal (t = 90 min) for glucose analysis. Glucose concentrations were analyzed as an indirect indicator for the site of delivery of the nutrients and subsequent nutrient absorption. Sodium fluoride tubes (Becton & Dickinson, NJ, USA) were used to collect blood samples for plasma glucose concentrations. Glucose measurements were performed using a Roche Cobas C701 analyzer (GLUC3, Roche, Mannheim, Germany) with an inter-assay variation of 0.02 mmoL/L at glucose concentration 3.27 mmoL/L.

### 2.9. Statistical Analyses

Statistical analyses were performed with SPSS, version 22.0 (SPSS Inc., Chicago, IL, USA). Statistical analysis of food intake was performed on the amount of food eaten in kcal. Plasma glucose concentrations and VAS scores are displayed from before the start of ingestion of test drink (t = 0 min) until the *ad libitum* meal (t = 90 min). All variables were compared with a mixed model analysis of variance, including the fixed factor treatment (encapsulated nutrient mixture and control). For the VAS scores and plasma glucose concentrations, time and the interaction between treatment and time were added to the model. To compare the intervention effects within subjects in this cross-over design, the subject was included as a random factor. The mixed model included a random intercept model and a random slope (variance components) model. The final model was chosen based on the Akaike Information Criterion (AIC); the model with the lowest AIC was suitable for interpretation. Total VAS scores and plasma glucose concentrations in the 90-min time frame after the ingestion of the test drink were expressed as the area under the curve (AUC), which was calculated with the use of the trapezoid rule and analyzed with the use of a paired *t* test. Data are presented as the means ± SD unless specified otherwise. *p* < 0.05 was considered as statistically significant.

## 3. Results

**Figure 2:**
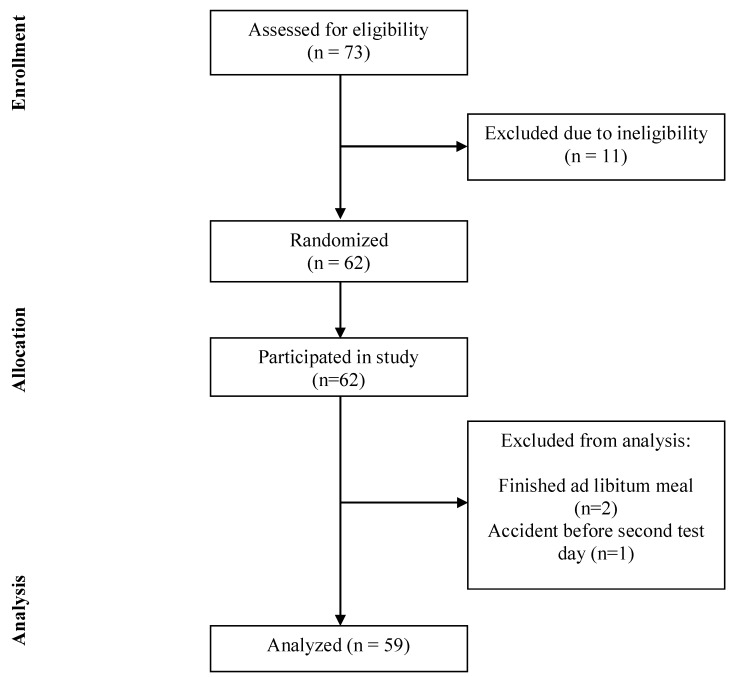
CONSORT flow diagram.

Sixty-two subjects were included in this study (21 male, age: 43.3 ± 15.9 years, BMI 27.8 ± 1.49 kg/m^2^). Two subjects were excluded from analysis, because we were not able to reliably measure their *ad libitum* intake of the test meal; since these subjects finished the whole meal without being satiated. One participant dropped out during the study protocol, because of a traffic accident the day before the test day (Figure 2).

### 3.1. Food Intake

**Figure 3:**
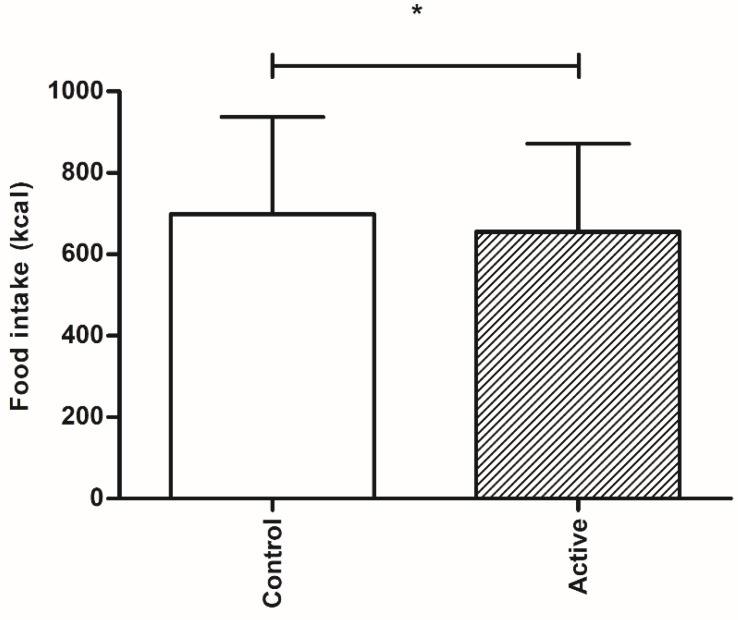
Food intake in kcal (mean ± SD) of the *ad libitum* meal offered 90 min after the ingestion of the active or control product. * *p* < 0.05.

Ingestion of an encapsulated nutrient mixture resulted in a significantly lower food intake during the *ad libitum* meal compared with control (655 ± 30 kcal compared with 699 ± 30 kcal respectively, *p* = 0.011, Figure 3).

### 3.2. Satiety, Hunger, Fullness and Desire to Eat Scores

**Figure 4:**
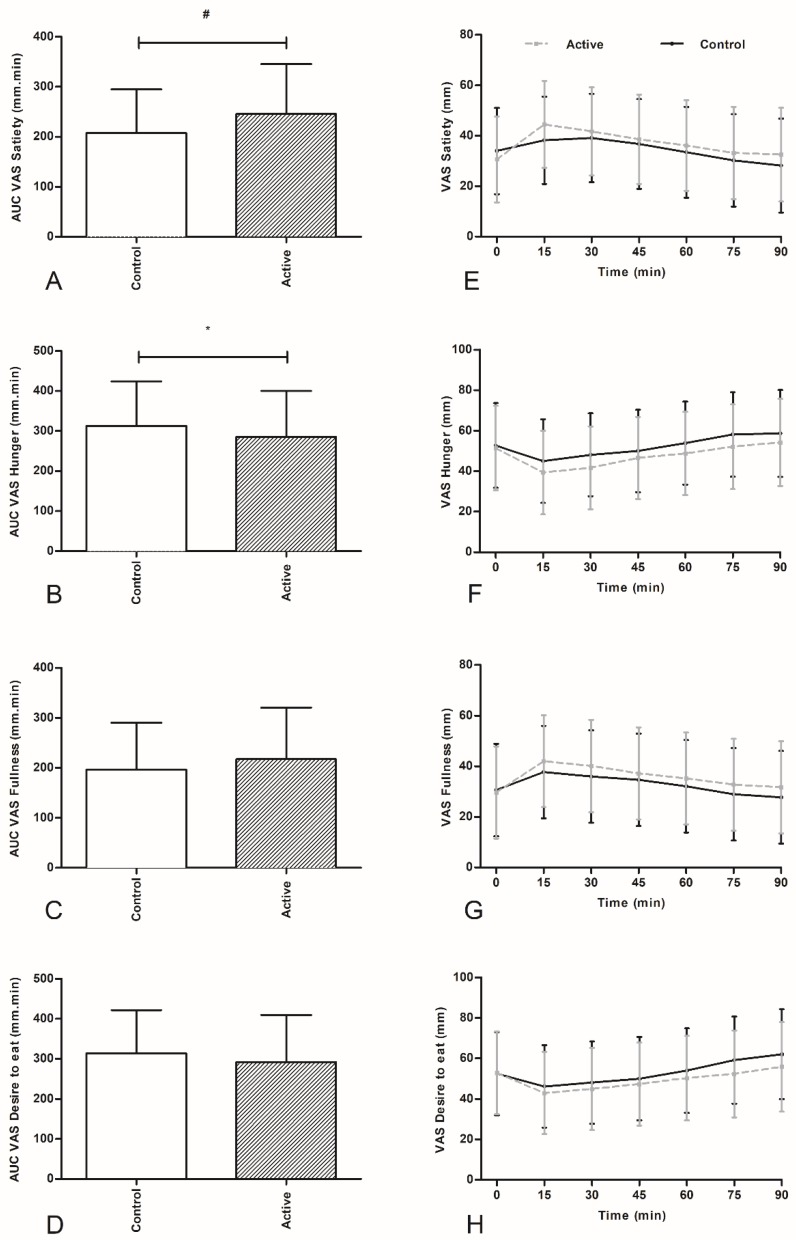
AUCs (0–90 min) for Satiety (**A**), Hunger (**B**), Fullness (**C**) and Desire to eat (**D**) (mean ± SD). VAS scores for Satiety (**E**), Hunger (**F**), Fullness (**G**), and Desire to eat (**H**) after intake of active or control product (mean ± SD). Ingestion of the test drink took place at t = 0 min (180 min after consumption of the breakfast). An *ad libitum* meal was offered at t = 90 min. AUCs were calculated by using the trapezoid rule. * *p* < 0.05 and ^#^ *p* < 0.01.

Ingestion of both products decreased hunger and desire to eat and increased satiety and fullness. While this effect was more pronounced for the active product for all VAS parameters, there was no significant treatment x time interaction observed for all the VAS parameters. With respect to the AUC (from 0 to 90 min), significant differences between the active and control product were observed for satiety (*p* < 0.01) and for hunger (*p* < 0.05) (Figure 4).

### 3.3. GI Symptoms

Mean scores for pain, bloating, flatulence, heartburn, nausea, belching, cramps and urge to defecate did not differ between the ingestion of the encapsulated nutrient mixture and the control. Subjects did not experience any GI symptoms after ingestion of both treatments.

### 3.4. Glucose Concentrations

**Figure 5:**
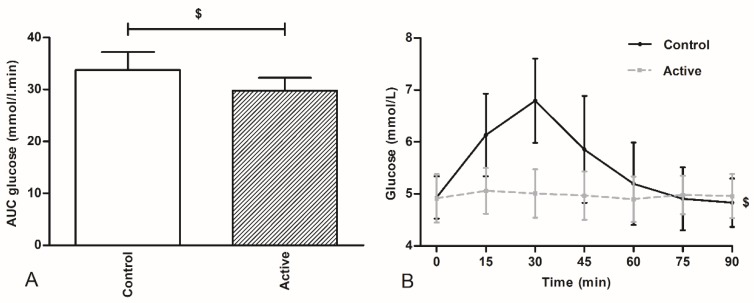
AUC of the plasma glucose concentration (mean ± SD), (**A**) and plasma glucose concentrations over time (mean ± SD), (**B**) during the period after ingestion of the test drink (active and control) scheduled from 0 to 90 min. AUCs were calculated by using the trapezoid rule. ^$^ *p* < 0.001.

The plasma glucose concentration before ingestion of the products (t = 0 min) did not differ between the test days (*p* > 0.05). There was a treatment × time interaction for plasma glucose concentrations; plasma glucose concentrations increased after ingestion of the control product over time, whereas the active product did not result in changes in plasma glucose concentrations, *p* < 0.001. A significant increase in AUC of the plasma glucose concentration was observed after the control compared with to the active product, *p* < 0.001 (Figure 5).

## 4. Discussion

In this proof of concept study, we have shown that oral ingestion of an encapsulated nutrient mixture of sucrose and casein, targeted at delivery into the more distal part of the intestine of healthy overweight individuals, resulted in a decreased caloric intake of a subsequent lunch intake when compared to the control product. The decrease in food intake was accompanied by increased feelings of satiety and decreased feelings of hunger.

The effect of an orally ingested encapsulated nutrient mixture to reduce food intake has not been evaluated previously in humans. Our data confirm that the concept of inhibition of food intake by encapsulated nutrients indeed is effective in an acute intervention experiment. Our test product induced significant effects on food intake and satiety after one single administration of the orally ingested product. This finding is promising for clinical applications, but needs confirmation in longer term, repetitive experiments. Previously van Avesaat et al., have investigated the effects of ileal infusion of protein and carbohydrate separately on the ileal brake targeting on food intake and satiety in normal weight subjects using a naso-ileal intubation technique [4]. It was shown that infusion of protein and carbohydrate in a total concentration of 51.7 kcal resulted in a decrease in food intake of respectively 128.7 kcal and 187.7 kcal [4]. We here observed a decrease in food intake of 44 kcal, an effect of considerably lesser magnitude. A possible explanation for the less pronounced effect on food intake in our current study may result from several factors. First, it may be caused by the method of nutrient delivery, resulting in differences in amounts of the macronutrients present in the more distal intestinal lumen during the study. Avesaat et al., infused 51.7 kcal of carbohydrate and protein separately over 90 min directly into the ileum. This resulted in a caloric load of 0.57 kcal/min. In our study protocol, subjects acutely and completely ingested both test drinks 90 min before start of the *ad libitum* meal. We are not informed about the amount of protein and carbohydrate calories present in the distal intestine (ileum and more distally) before start of the *ad libitum* meal in the present study, and thus on the magnitude of intestinal brake activation. Furthermore, we chose a combination of two macronutrients. Several studies have shown that infusion of a combination of different macronutrients in the duodenum did not result in synergic effects on food intake and satiety [18,23]. It is thought that a critical, minimum amount of amino acids and monosaccharides should be present in the intestinal lumen in order to reach a threshold value for activating the ileal brake [5]. One of the two macronutrients may not have reached the critical threshold level for intestinal brake activation. It should also be taken into account that not all of the encapsulated nutrients will have reached the ileum, either through more rapid and proximal delivery or through spill over into the colon. 

The effect of the ‘intestinal brake’ on food intake and satiety feelings has mainly been evaluated in normal weight subjects [24,25]. The effect of distal delivery of nutrients and the effectiveness of the ileal brake is less understood in overweight and obese subjects. For the duodenal brake, a less pronounced suppression of food intake was observed after intraduodenal lipid infusion in obese subjects compared to lean subjects [26,27]. According to our study protocol we included healthy overweight subjects with a BMI between 25 and 30 kg/m^2^ and observed a significant decrease in food intake. It is not known whether the effect of more distally delivered nutrients on food intake is different in overweight compared to normal weight subjects. On the other hand, several studies investigating the mechanisms of weight loss after bariatric surgery, such as Roux-en-Y gastric bypass, have provided clear evidence of increased delivery of nutrients in the jejunum and ileum, ileal brake activation and a sustained decrease in body weight years after intervention [28].

This study was explorative in design: our aim was to deliver proof for the concept that encapsulates nutrients, targeting at a more distal intestinal release, will inhibit subsequent caloric intake. Ileal brake activation is associated with more pronounced release of distal gut peptide into the systemic compartment. We have not measured gut peptide release, so proof for more distal gut delivery of the encapsulated protein-carbohydrates mixture cannot be delivered. However, we measured plasma glucose concentrations at regular intervals for 90 min after ingestion of the encapsulated mixture and after ingestion of the non-encapsulated identical mixture. While plasma glucose concentrations increased significantly within thirty min after ingestion of the non-encapsulated product, this increase was not found after ingestion of the encapsulated nutrient mixture, pointing to at least delayed, more gradual and possibly more distal delivery the sucrose from the encapsulate. 

Several limitations of this study should be acknowledged. First, our study was designed to deliver evidence for the proof of concept, providing first in-human data whether an encapsulated nutrient mixture is able to reduce food intake in an acute intervention setting. Second, we have not measured timing or location of release of nutrients from the encapsulate into the lumen nor have we measured distal gut peptide like PYY and GLP-1 to obtain further insight whether the more distal part of the small intestine indeed was activated. Subsequent studies are needed to deliver further proof of ileal brake activation. Third, we evaluated food intake in the acute setting, which is after intake of only a single ingestion of the encapsulated nutrient mix. Fourthly, it is not known whether encapsulated nutrients are able to repetitively activate ileal brake mechanisms and hereby result in a sustained effect on food intake and satiety. Due to the encapsulation material, the encapsulated nutrient mixture consisted of slightly more calories than the control product. It is however not expected that this small difference would have affected the results. Increasing the number of calories in the test drink would lead to more potent ileal brake activation and consequently a more pronounced effect on the inhibition on food intake. While the decrease in food intake in terms of calories appears rather modest after a single use, future studies should reveal whether this effect is sustained after repetitive ingestion of this encapsulated nutrient mixture. Another focus for future studies is on the exact behavior of the encapsulated nutrient mixture in the GI tract, to provide proof of ileal brake activation. The question needs to be answered whether such a slow release, repetitive ingestion strategy will have a potential role in long term weight management.

In conclusion, this is the first in human study to demonstrate that ingestion of an encapsulated protein and carbohydrates mix targeting the distal small intestine is able to decrease food intake and increase satiety in overweight subjects in an acute setting.
